# Toe digit pulsation measurement by impedance plethysmography in miniaturized devices: An early feasibility test

**DOI:** 10.2478/joeb-2025-0020

**Published:** 2025-12-31

**Authors:** Håvard Kalvøy, Jonny Hisdal, Christian Tronstad

**Affiliations:** Department of Clinical & Biomedical Eng, Oslo University Hospital,Norway; Faculty of Medicine, University of Oslo,Norway; Department of Vascular Surgery, Oslo University Hospital,Norway

**Keywords:** Bioimpedance, plethysmography, toe, artery, tetra-polar, flexible electrodes, pulsatile blood flow

## Abstract

This early feasibility test explores impedance plethysmography (IPG) for detecting toe pulse waves using a setup suited for miniaturized devices. Conventional tests often miss micro-circulatory impairment, which is critical for wound healing and limb survival. We designed a flexible 3D-printed clamp with Ag/AgCl electrodes positioned on the inner big toe to target the lateral plantar digital artery. 50 kHz impedance measurements were performed on a healthy volunteer using 3D-printed electrode clamp for a tetrapolar configuration. Custom designed flexible electrodes were tested with and without contact gel. Preliminary results suggest that IPG can capture distal pulsatile signals, motivating further exploration of the potential for early detection of peripheral vascular dysfunction.

## Introduction

Peripheral arterial disease (PAD) and diabetic neuropathy are major contributors to chronic limb ischemia, tissue breakdown, and in worst case amputation [1]. Conventional diagnostic approaches, such as ankle-brachial index (ABI), toe pressures, and angiography, provide valuable information about macrovascular patency but often fail to capture microcirculatory status and functional perfusion at the distal extremities [2]. This gap is critical because impaired microcirculation and tissue perfusion strongly influence wound healing, neuropathic complications, and limb survival.

Four-electrode measurement of bioimpedance is well known to be sensitive to pulsations from the aorta (impedance cardiography) and different levels of the arterial tree down to peripheral locations such as the arm, wrist and legs. The concept of impedance plethysmography (IPG) was introduced in the early 1900s and after some decades there were numerous suggestions for distal applications in extremities such as lover arm, fingers, legs, feet and toes [3,4,5,6]. Integration in wristwatches [7] and smart-socks [8] are investigated in more recent work, and IPG in peripheral arteries can also be used to obtain pulse wave velocities [9].

Carter & Tate [10] suggested that waveform analysis from distal hemodynamic events adds prognostic information beyond static pressure indices and reported that they from toe pulse wave amplitude measured by photoplethysmography could point out risk factors strongly associated with major amputation and mortality in patients with PAD and skin lesions. An analyzable waveform of pulsatile changes in peripheral blood volume from the toe will represent the most distal measurement possible and probably be a sensitive and early marker for vascular dysfunction and diagnostic of peripheral arterial disease. We here present some early results from a pilot investigation of IPG measurement on the inner side of the big toe targeting the lateral plantar digital artery on the foot of a healthy volunteer. Because, to our knowledge no prior IPG measurements have been reported for this artery, we evaluated whether a non-invasive, rapid IPG assessment could yield information on distal blood flow dynamics and serve as an early signal of compromised peripheral circulation.

## Materials and methods

**Figure 1.**
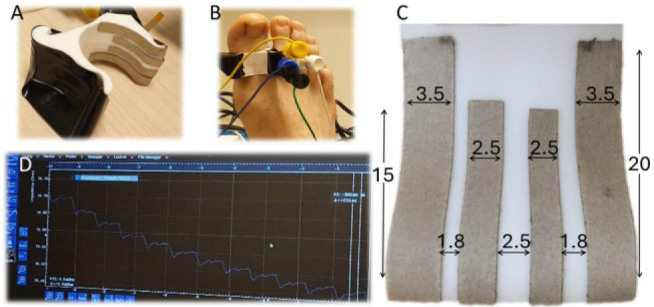
A: 3D-printed clamp with electrodes. B: Measurement position on the inside of right great toe. C: Electrode setup (approx. area CC = 70 mm^2^, PU = 37,5 mm^2^) D: Measurement software on the impedance analyzer.

An electrode support designed to weakly clamp non-adhesive electrodes to the skin was 3D-printed using a flexible material (thermoplastic polyurethane, shore hardness 95A– fig. 1A). Strips of flexible electrodes were manufactured using Ag/AgCl ink (126-49(SP)C, Conductive Materials, MA, USA) heat-cured on a polyimide substrate. The strips were glued to the inner surface of the supporting electrode clamp following the round shape in contact with skin at the lateral side of the toe (fig. 1B). Connection pads for measurement leads were obtained on the outside of the support by wrapping the strips around the end to allow connection by hook test leads. The contact area of the electrode strips was together with the width of the strip, determined by the length of the strip that was in electric contact with the skin before bending away to the outside. This gave a contact area of 70 mm^2^ (3.5 mm × 20 mm) for the outermost current-carrying electrodes, and 37,5 mm^2^ (2.5 mm × 15 mm) for the inner pair of pick-up electrodes (fig. 1C). The distance between the electrode pairs was 1.8 mm and the gap between the inner strips was 2.5 mm (fig. 1D). The tetrapolar electrode arrangement was placed on the right foot of a healthy volunteer to cover the expected anatomical trajectory of the lateral plantar digital artery.

**Figure 2.**
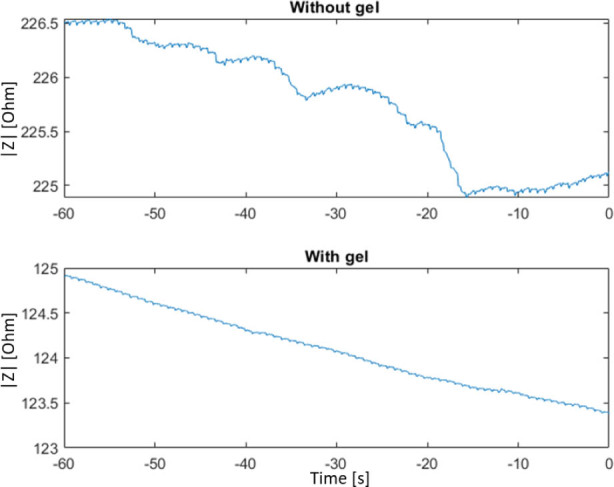
Example raw data measurements over 1 minute with the toe probe without and with gel applied to the electrode surface.

**Figure 3.**
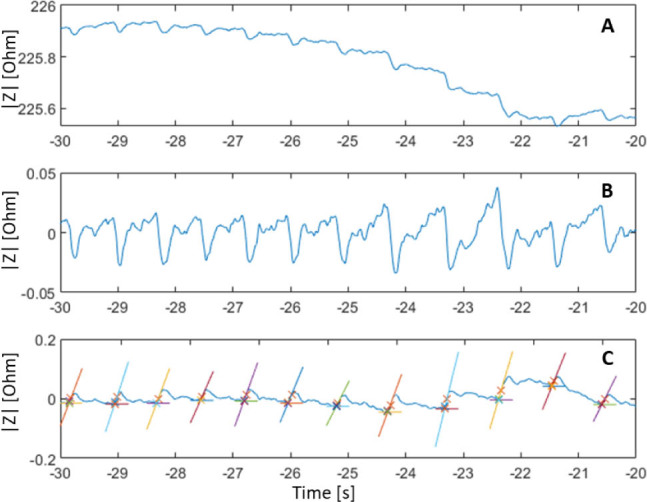
Signal processing of a 10 s segment of the without gel measurement showing A: raw data, B: high-pass filtered signal and C: identification of upstroke fiducial points based on the intersecting tangents method plotted along with the tangents on a negated filtered signal.

A 50 kHz and 300 mV excitation signal was used to sample the impedance at a rate of 838 Hz (MFIA, Precision LCR Meter 5 MHz, Zürich Instruments, Switzerland, Fig 1D). The first recording was done with dry electrodes and then repeated after careful application of contact gel (Spectra 360 Gel, Parker Laboratories Inc, US) on the electrode surfaces (Fig. 2). The raw data was high-pass filtered and upstroke fiducial points were identified based on the intersecting tangents method [9, 11] (Fig. 3).

### Informed consent

Informed consent has been obtained from the individual included in this study.

### Ethical approval

Non-invasive data captured for illustrative purposes, as a test of the measurement method, is exempt from the requirement for ethical approval according to Norwegian law.

## Results

**Figure 4.**
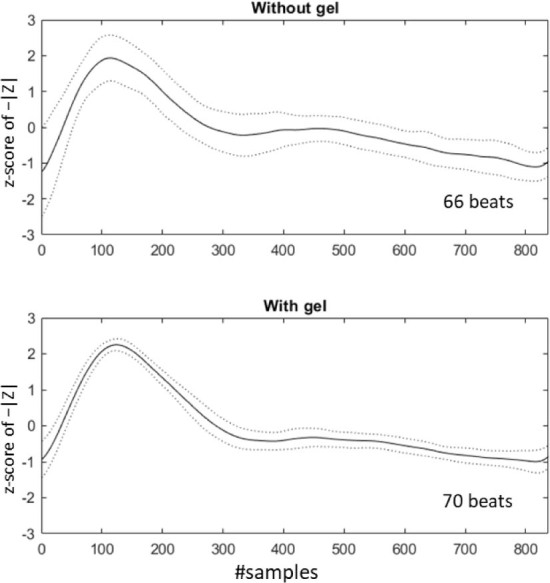
1-minute ensemble averaged IPG waveforms of the normalized example measurements without and with gel. The solid lines show the mean over all pulse waveforms and the dashed lines show the mean ± standard deviation.

We found distinct IPG waveform curves both with and without gel, but Figure 4 clearly shows that the application of gel gave significantly more stable measurement with less noise and smoother recognizable pulse waveforms (less variability between pulses as indicated by the standard deviations).

## Discussion

Our plethysmographic measurement example shows a waveform that correlates in morphology to what we expected. But we also experienced that the measurement was dependent on small changes in electrode position and the pressure against the skin. At the same time, we do not know the exact origin of the observed measurement result. It is known that IPG is in general more sensitive to deeper vessels (arteries) and less sensitive to superficial perfusion especially compared to PPG. However, this depends on electrode geometry and anatomical location. It is therefore not known [12] which signal sources are most dominant, and which are confounding; the artery, arterioles, capillaries, or potentially compression / decompression of dermal tissue [13]. Anyhow, we were dependent on a delicately designed clamp to balance the contact pressure on the skin, to not obstruct the blood flow we aim to measure while avoiding variable electric contact.

We designed the electrode setup for transversal measurement with a relatively long electrode to allow sensitivity to variable anatomical locations of the artery, but from theory we might assume that higher sensitivity could be obtained by focusing the measurement zone between the middle (voltage pickup) electrodes alongside the artery. From this initial testing we learned that it was very challenging to obtain a reliable and reproducible position due to thin arteries. We also expect challenges due to interindividual anatomical differences, especially for a longitudinal electrode arrangement.

At the very least, this IPG measurement is suitable for pulse detection and heart rate estimation, while pulse wave analysis will likely require an improvement in signal quality, especially for dry electrodes. While we report feasibility of IPG measurement at this location, there are challenges to be addressed including: Signal quality, waveform reproducibility, reduction and stability of electrode contact impedance, and contact pressure optimization. Future work also includes optimization of electrode geometry, comparison on different subjects for anatomical variability, comparison to reference measurements such as toe pressure and photoplethysmography, and the suitability of the sensing location in pulse wave velocity measurement.
